# A spatiotemporal transformer with cross-frame encoding and trajectory-aware decoding for multi-target fish tracking

**DOI:** 10.1038/s41598-025-31686-8

**Published:** 2025-12-10

**Authors:** Yang Li, Lei Han

**Affiliations:** Heilongjiang Province Hydraulic Research Institute, Harbin, China

**Keywords:** Multi-target tracking, Underwater vision, Spatiotemporal transformer, Trajectory perception decoding, Engineering, Mathematics and computing

## Abstract

In response to the challenges of multi-object fish tracking in complex underwater environments, where performance is easily affected by illumination changes, suspended particles, occlusion, and high inter-target visual similarity, this paper proposes a unified Transformer framework that integrates cross-frame spatiotemporal encoding with trajectory-aware decoding. In the encoding stage, temporal difference and frame position embeddings are introduced and combined with a residual motion enhancement mechanism to explicitly align appearance, scale, and displacement across frames. In the decoding stage, trajectory extrapolation priors and temporal association attention are employed to restrict cross-frame feature aggregation within reasonable candidate regions, achieving joint optimization of detection and association. On our self-constructed underwater fish tracking dataset, the proposed method achieves MOTA, IDF1, and Recall scores of 0.719, 0.693, and 0.742, improving over the strong baseline model GTR (0.688, 0.671, 0.720) by 0.031, 0.022, and 0.022 absolute points. On the UOT32 dataset, it attains 0.697, 0.680, and 0.730, surpassing ByteTrack (0.675, 0.650, 0.700) by 0.022, 0.030, and 0.030 absolute points, respectively. These results demonstrate that the proposed approach effectively integrates cross-frame spatiotemporal modeling with trajectory-guided decoding, enabling accurate detection and reliable identity association even under occlusion and dense target conditions. The method exhibits strong robustness and generalization in complex underwater environments, outperforming existing state-of-the-art approaches in both tracking accuracy and stability.

## Introduction

Multi-object fish tracking technology holds significant application value in fields such as aquaculture, fishery resource monitoring, fish passage assessment, and underwater ecological behavior research^1^. By achieving accurate detection and continuous tracking of individual fish within a school, researchers and industry practitioners can estimate population size, evaluate migration efficiency in fishways, assess individual health conditions, and analyze behavioral patterns. Such capabilities provide a scientific basis for intelligent farming management, disease prevention, ecological conservation, and the design of more effective fish passage facilities^2^.

In complex underwater environments, fish often exhibit dense distributions, rapid movements, and highly variable postures, which impose stringent requirements on the robustness and real-time performance of multi-object tracking algorithms^3^. However, existing methods still face numerous challenges. First, illumination changes, suspended particles, and water surface reflections degrade image quality, making target detection susceptible to interference. Second, frequent occlusions among fish and their high visual similarity often lead to errors in cross-frame identity association, resulting in drift and ID switching^4^. Third, many tracking frameworks designed for generic scenarios struggle to effectively model global motion patterns over long temporal sequences, leading to suboptimal performance in complex motion and trajectory prediction tasks. Furthermore, some approaches fail to fully exploit temporal information and motion priors, causing a disconnect between the detection and association stages, which adversely affects overall performance^5^.

To address these issues, this paper proposes a unified Transformer-based framework with cross-frame spatiotemporal encoding and trajectory-aware decoding. On the encoding side, we introduce a joint embedding of temporal difference and frame position information, combined with a residual motion enhancement mechanism, to explicitly align appearance, scale, and displacement across frames. On the decoding side, we employ trajectory extrapolation to generate spatial priors, together with a temporal association attention mechanism, to restrict cross-frame feature aggregation within reasonable candidate regions, thus achieving joint optimization of detection and identity preservation. This method not only enhances robustness under occlusion and illumination variations but also effectively reduces drift and ID switching in long-sequence tracking, thereby supporting reliable monitoring of fish schools in natural habitats and engineered environments such as fishways. This article also provides a comparison table with other Transformer architecture methods, as shown in Table 1.

**Table 1:** Comparison of core differences with existing transformer-based tracking methods.

Method	Modeling Paradigm	Spatiotemporal Information Utilization	Association Constraint Mechanism	Main Innovation
TrackFormer^6^	End-to-end joint modeling of detection and association based on DETR	Single-frame feature encoding + temporal query propagation	Attention-based implicit matching without motion prior	Unified framework but insufficient for long-term motion modeling
MOTR^7^	Transformer-level joint learning for detection and tracking	Multi-frame feature fusion with shallow temporal modeling	Dynamic query initialization without explicit trajectory extrapolation	Emphasizes end-to-end reasoning but unstable under occlusion
ViViT^8^	Video-level Transformer architecture	Global spatiotemporal attention with implicit frame relations	No explicit association mechanism	Strong spatiotemporal awareness but unsuitable for target-level association tasks
**Ours**	Unified cross-frame spatiotemporal encoding + trajectory-aware decoding framework	Cross-frame differential embedding + residual motion enhancement for explicit motion alignment	Incorporates trajectory extrapolation priors and temporal association attention to explicitly constrain association range	Enhances long-term motion modeling and occlusion robustness, adapted to complex underwater environments

The main contributions of this work are as follows:

- We propose a cross-frame spatiotemporal encoding strategy that fuses appearance, displacement, and temporal information within a unified feature space, enhancing the model’s long-term motion perception and occlusion recovery capabilities.
- We design a trajectory-aware decoding module with a temporal association attention mechanism, which leverages extrapolated trajectory priors and candidate region masking to effectively constrain the cross-frame association range, improving the consistency between detection and association.
- We conduct comprehensive experiments on a self-constructed underwater fish tracking dataset and the UOT32 dataset, demonstrating the superiority of our method in multiple metrics, including MOTA, IDF1, and Recall.
- Through trajectory overlay and Grad-CAM visualization, we illustrate the model’s ability to focus on key regions and capture motion directions in complex underwater scenes, further enhancing the interpretability of the method.

## Related work

### Visual transformer related research

Visual Transformers have rapidly emerged as a prominent research direction in computer vision since the introduction of the Vision Transformer (ViT) model by Dosovitskiy et al.^9^. This approach partitions an image into fixed-size patches and maps them into a sequence for Transformer processing, enabling global feature modeling without convolutional operations. Subsequently, Touvron et al.^10^ proposed DeiT, which significantly reduced the training cost of ViT by introducing a data-efficient distillation mechanism; Liu et al.^11^ introduced the Swin Transformer, employing a shifted window mechanism to achieve hierarchical feature extraction that balances computational efficiency and multi-scale modeling capability; Wang et al.^12^ proposed the Pyramid Vision Transformer (PVT) and Wu et al.^13^ developed the Convolutional Vision Transformer (CvT), both of which integrate the strengths of convolution and Transformer architectures to enhance performance in dense prediction tasks. More recently, He et al.^14^ proposed Masked Autoencoders (MAE) and Oquab et al.^15^ developed DINOv2, both demonstrating superior generalization in self-supervised visual representation learning and providing high-quality features for downstream tasks.

In the domain of video understanding, the spatiotemporal modeling capabilities of Transformers have been extensively validated. Bertasius et al.^16^ introduced TimeSformer, which employs a space-time factorized attention mechanism to efficiently capture dynamic information in videos; Arnab et al.^8^ proposed ViViT, exploring various spatiotemporal attention architectures for video feature encoding; Liu et al.^17^ extended the Swin architecture to the video domain with the Video Swin Transformer, achieving state-of-the-art performance in video action recognition and video object detection. These methods have effectively improved cross-frame feature association, providing methodological foundations for cross-frame encoding in multi-object tracking tasks.

In the field of object detection, Carion et al.^18^ pioneered the use of Transformers for end-to-end object detection with DETR, which eliminates handcrafted components of traditional detectors through set-based prediction; more recently, Zhao et al.^19^ proposed RT-DETR, achieving real-time inference while maintaining detection accuracy, thus offering a promising solution for detection and tracking in real-time scenarios. Collectively, these advances form a solid technical foundation for the proposed framework that integrates cross-frame encoding and trajectory-aware decoding for multi-object fish detection and tracking.

### Research on target tracking algorithms

#### Single target tracking algorithm

Single Object Tracking (SOT) aims to accurately and efficiently predict the location of a given target in subsequent video frames, given its initial position in the first frame. Early methods primarily relied on similarity matching based on convolutional features. Bertinetto et al.^20^ first applied a fully convolutional Siamese network to visual tracking in SiamFC, achieving an end-to-end framework for feature extraction and matching. Li et al.^21^ introduced the Region Proposal Network (RPN) into the Siamese architecture with SiamRPN, effectively improving localization accuracy and bounding box regression. SiamRPN++^22^ further incorporated deeper backbone networks and multi-scale feature fusion strategies, achieving a better balance between accuracy and speed. In addition, Zhang et al.^23^ proposed the Structured Siamese Network (StructSiam), which enhanced matching robustness through structured feature modeling.

To further improve discriminative capability and adaptability, researchers have incorporated online updating and discriminative model prediction mechanisms. Danelljan et al.^24^ proposed ATOM, which integrates IoU prediction with a classification branch and achieves superior localization accuracy. Bhat et al.^25^ introduced DiMP, which learns a generalizable discriminative model predictor with stronger adaptability across diverse scenarios. Wang et al.^26^ explored the integration of natural language and visual tracking, constructing multimodal benchmarks and algorithms that support both target localization and semantic conditional constraints, thus opening new research directions for interactivity and flexibility in tracking.

In recent years, Transformer architectures have been introduced into SOT, significantly enhancing cross-frame feature modeling. Chen et al.^27^ proposed TransT, which leverages attention mechanisms to fuse template and search region features, thereby improving the discriminative power of feature representations. Yan et al.^28^ developed STARK, which models spatial and temporal dependencies simultaneously through a spatiotemporal Transformer, enabling end-to-end tracking prediction. Chen et al.^29^ further introduced the “Backbone is All You Need” simplified architecture, which employs an efficient backbone network to substantially reduce computational complexity while maintaining accuracy. More recently, Hoanh and Pham^30^ proposed a density-guided query selection strategy to enhance Transformer-based detection of small objects, while Than et al.^31^ introduced a long-range feature aggregation and occlusion-aware attention mechanism to improve detection robustness in autonomous driving scenarios. These studies collectively provide a solid foundation for subsequent tracking frameworks that integrate spatiotemporal information with efficient decoding mechanisms.

Moreover, since the proposed framework demonstrates strong adaptability across diverse underwater environments, it also shows potential for future extension to domain adaptation tasks, where models trained on one underwater scene can generalize to others with different visual domains. Related research on semi-supervised and multi-source domain adaptation provides valuable references for this direction, such as Kim et al.^32^, Ngo et al.^33^, and Ngo et al.^34^, which explore domain-specific knowledge distillation, trico-training strategies, and divide-and-conquer approaches for robust cross-domain generalization.

#### Multi-target tracking algorithm

Multi-Object Tracking (MOT) aims to simultaneously localize multiple targets in a video sequence while maintaining consistent identities over time, and is commonly implemented under the tracking-by-detection paradigm. Representative early works include SORT^35^, which achieves efficient online tracking via Kalman filtering and the Hungarian matching algorithm; Deep SORT^36^ extends this framework by incorporating deep appearance features for similarity measurement, significantly improving identity preservation under occlusions and appearance variations. Subsequently, CenterTrack^37^ integrates object detection and short-term association into a single-stage prediction framework, reducing intermediate processing steps, while FairMOT^38^ further addresses the imbalance between detection and re-identification (Re-ID) branches in traditional pipelines by jointly optimizing both tasks within a unified network.

With the growing application of Transformers in vision tasks, researchers have explored their potential for MOT. Sun et al.^39^ first introduced Transformers into MOT with TransTrack, jointly modeling detection results and tracking queries through an encoder-decoder structure. Meinhardt et al.^6^ proposed TrackFormer, which unifies object detection and trajectory association within a DETR-style end-to-end framework, eliminating the need for post-hoc association. Zeng et al.^7^ developed MOTR, which employs a query dynamic updating mechanism to maintain consistent identities across frames, thereby enhancing long-term tracking capability. Xu et al.^40^ proposed TransCenter, which leverages dense representations to improve robustness in crowded scenes.

In recent years, MOT algorithms have made substantial progress in both robustness and real-time performance. Zhang et al.^41^ proposed ByteTrack, which improves recall by associating all detected bounding boxes, including those with low confidence, while maintaining high precision. Cao et al.^42^ introduced Observation-Centric SORT, which adopts an observation-driven matching strategy to achieve stable performance under occlusion and missing detection conditions. Luiten et al.^43^ proposed Track to Reconstruct, which combines three-dimensional reconstruction with tracking, exploiting spatiotemporal consistency to improve overall tracking accuracy. Collectively, these studies provide a solid technical foundation for integrating spatiotemporal Transformers with real-time detection models, such as RT-DETR, to achieve high-precision multi-object tracking. Moreover, recent advances in graph-based and knowledge distillation approaches, such as HiGDA^44^ and Cross-domain Knowledge Distillation^45^, offer promising directions for enhancing domain adaptability and representation generalization, which could inspire future extensions of our framework toward cross-domain underwater tracking scenarios.

## Method

### Overall model architecture

**Fig. 1:**
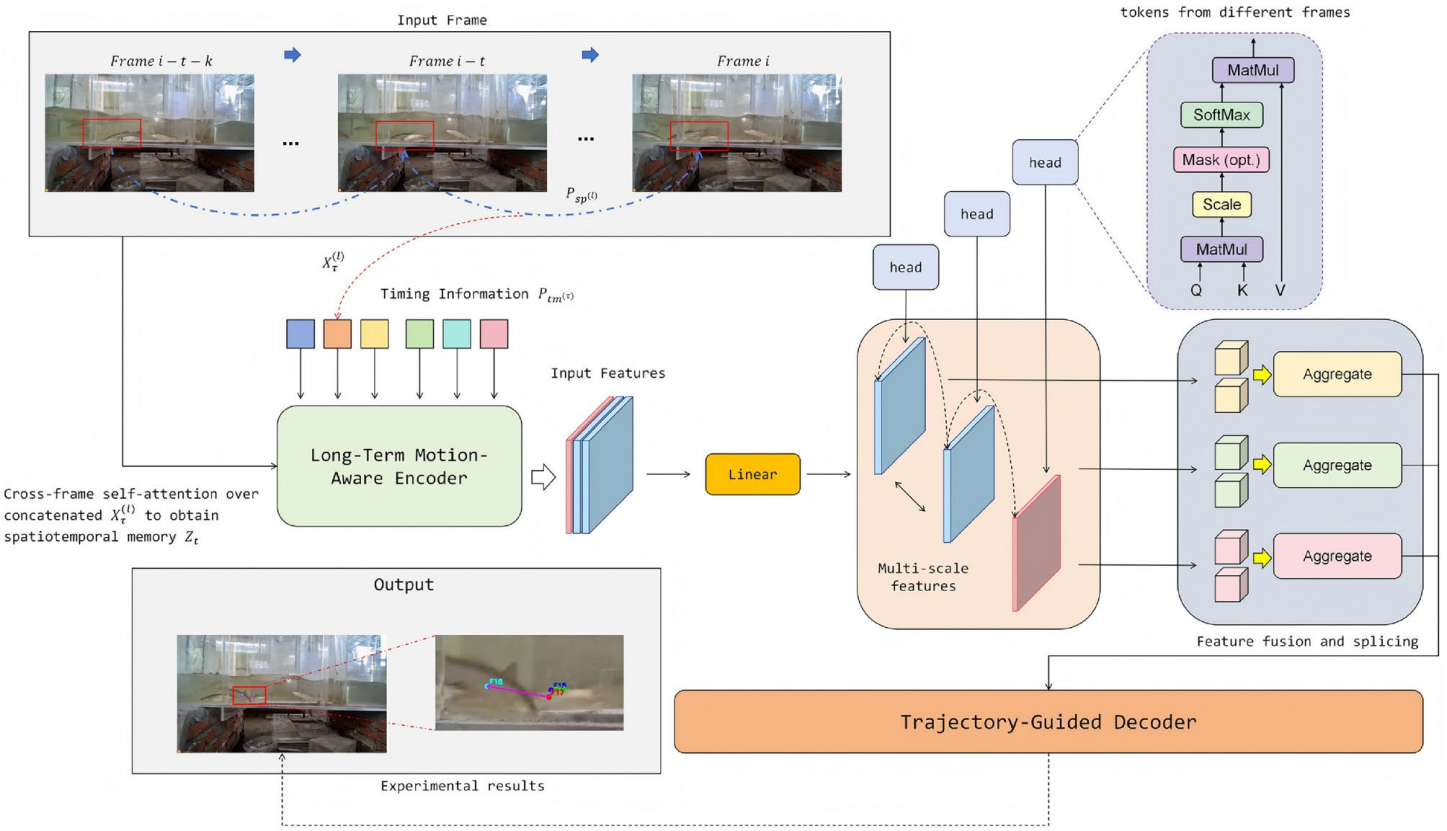
Overall framework structure. The model employs a long-term motion-aware encoder to fuse cross-frame temporal information with multi-scale features, and leverages a trajectory-guided decoder to achieve joint target detection and association within a unified spatiotemporal modeling framework.

This study addresses the task of multi-object fish tracking. Given a video sequence $\{\textbf{I}_t\}_{t=1}^{T}$, the objective is to output, for each frame *t*, a set of targets $\mathscr {Y}_t=\{y_t^i\}_{i=1}^{N_t}$, where $y_t^i=(\textbf{b}_t^i, \ell _t^i, \textrm{id}_t^i)$ denotes the bounding box parameters (center, scale, or four vertices), the class label, and the identity identifier, respectively. The overall architecture employs a real-time detector as the backbone, integrating cross-frame encoding and trajectory-aware decoding to enable end-to-end set prediction. The overall model architecture is shown in Fig 1.

First, a multi-scale backbone network extracts features ${\textbf{F}t^{(l)}}{l=1}^{L_s}$; for each scale, the features are partitioned into patches and linearly projected into tokens, to which spatial positional encodings $\textbf{P}{\textrm{sp}}^{(l)}$ and temporal encodings $\textbf{P}{\textrm{tm}}(t)$ are added, forming the cross-frame input sequence. The formula is as follows:

1 $$\begin{aligned} \textbf{X}{\tau }^{(l)}=\Pi !\left( \textbf{F}{\tau }^{(l)}\right) +\textbf{P}{\textrm{sp}}^{(l)}+\textbf{P}{\textrm{tm}}(\tau ) \quad \tau \in [t-L+1,t] \end{aligned}$$

where $\Pi (\cdot )$ denotes flattening and linear projection and *L* is the temporal window length. Here, $\textbf{P}{\textrm{sp}}^{(l)}$ is the scale-specific spatial positional encoding added to each token at scale *l*, and $\textbf{P}{\textrm{tm}}(\tau )$ is the temporal positional encoding that injects the frame index $\tau$ into the token representation to preserve temporal order.

A long-term motion-aware encoder operates on $\bigcup _{\tau =t-L+1}^{t}\bigcup _{l=1}^{L_s}\textbf{X}{\tau }^{(l)}$ via cross-frame self-attention and multi-scale interaction, producing a spatiotemporal memory representation, The formula is as follows:

2 $$\begin{aligned} \textbf{Z}t=\mathscr {E}\theta !\left( \bigcup {\tau =t-L+1}^{t}\bigcup _{l=1}^{L_s}\textbf{X}{\tau }^{(l)}\right) \end{aligned}$$

which explicitly aligns appearance, scale, and displacement information across frames within a unified latent space. This memory retains multi-scale contextual information essential for detection while encoding temporal motion patterns, thereby providing a unified spatiotemporal feature foundation for subsequent decoding. Here, $\mathscr {E}\theta (\cdot )$ denotes the encoder with learnable parameters $\theta$, and $\bigcup$ indicates concatenation/aggregation of tokens across the frames $\tau \in [t-L+1,t]$ and scales $l\in {1,\dots ,L_s}$ before attention-based fusion.

On the decoding side, the model maintains the trajectory set from the previous frame $\mathscr {T}{t-1}={(\textbf{b}{t-1}^j,\textrm{id}^j,\textbf{h}{t-1}^j)}{j=1}^{M_{t-1}}$, where $\textbf{h}{t-1}^j$ denotes the trajectory hidden state. Based on the historical position sequence $\mathscr {P}j={\textbf{b}{t-k}^j}{k=1}^{K}$, motion priors are computed and extrapolated as, The reasoning is as follows:

3 $$\begin{aligned} \tilde{\textbf{c}}{t|t-1}^j=\textbf{c}{t-1}^j+\big (\textbf{c}{t-1}^j-\textbf{c}{t-2}^j\big ) \qquad \tilde{\textbf{s}}{t|t-1}^j=\textbf{s}{t-1}^j \end{aligned}$$

from which trajectory-guided queries are constructed:

4 $$\begin{aligned} \textbf{q}t^j=\textbf{W}q!\left[ \textrm{PE}!\big (\tilde{\textbf{b}}{t|t-1}^j\big )\ \oplus \ g(\mathscr {P}j,\textbf{h}{t-1}^j)\right] \end{aligned}$$

and combined with a set of empty queries for discovering new targets to form $\mathscr {Q}t$. Here, $\textbf{c}{t-1}^j-\textbf{c}{t-2}^j$ is the frame-to-frame displacement (the minus sign denotes subtraction of the two most recent centers to estimate velocity under a constant-velocity prior), $\tilde{\textbf{c}}{t|t-1}^j$ is the extrapolated center at time *t*, and $\tilde{\textbf{s}}{t|t-1}^j$ keeps the previous scale $\textbf{s}{t-1}^j$ unchanged. In addition, $\textrm{PE}(\cdot )$ encodes the extrapolated box $\tilde{\textbf{b}}{t|t-1}^j$, $g(\cdot )$ fuses the historical positions $\mathscr {P}j$ with the hidden state $\textbf{h}{t-1}^j$, $\oplus$ denotes vector concatenation, and $\textbf{W}_q$ is a learnable projection that maps the concatenated features to the query space.

The trajectory-aware decoder takes $\textbf{Z}_t$ as keys and values and $\mathscr {Q}_t$ as queries, applying cross-attention and feed-forward updates to produce the set prediction:

5 $$\begin{aligned} \mathscr {Y}t=\mathscr {D}\phi (\mathscr {Q}_t, \textbf{Z}_t)={(\hat{\textbf{b}}_t^i,\hat{\ell }_t^i,\widehat{\textrm{id}}{}t^i)}{i=1}^{N_t} \end{aligned}$$

which is then used in a one-to-one set assignment and identity inheritance rule to update the trajectory set $\mathscr {T}t=\Psi (\mathscr {T}{t-1},\mathscr {Y}t)$. Here, $\mathscr {D}\phi (\cdot )$ denotes the decoder with parameters $\phi$, $\hat{\textbf{b}}_t^i$ is the predicted bounding box (center and size), $\hat{\ell }_t^i$ is the class label, $\widehat{\textrm{id}}{}_t^i$ is the predicted identity, and $\Psi (\cdot )$ updates trajectories by matching predictions to prior tracks with a one-to-one assignment.

The coupling of cross-frame encoding and trajectory-guided decoding enables detection and association to benefit jointly from the same attention computation: the encoder aggregates and aligns features across frames in space and time, while the decoder leverages intrinsic motion priors to guide target queries toward the correct instances, thereby maintaining stable output ${\mathscr {Y}t}{t=1}^{T}$ even in underwater scenes characterized by crowding, occlusion, and scale variation.

### Cross-frame temporal encoding in transformer for long-term motion awareness transformer

**Fig. 2:**
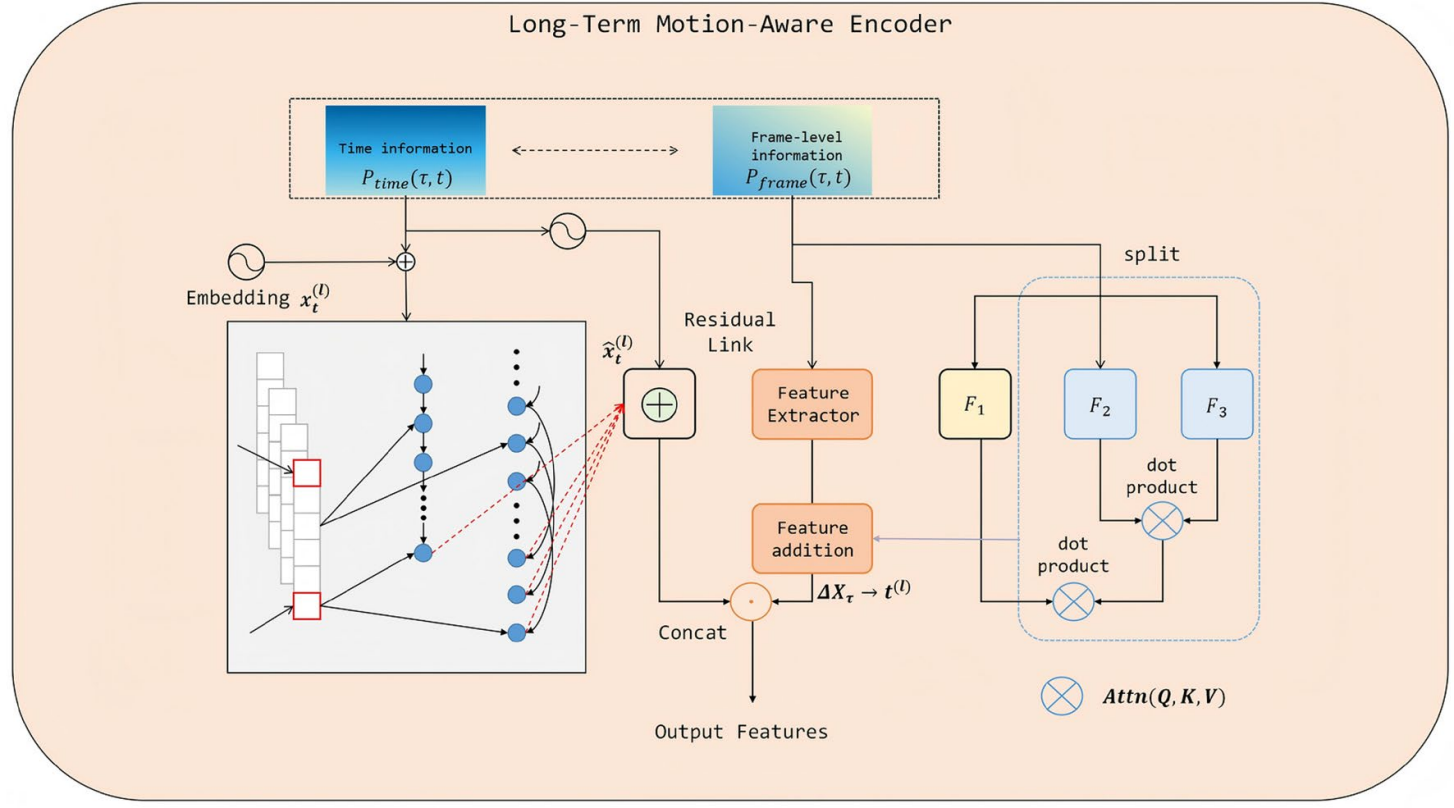
The schematic illustrates the cross-frame temporal feature modeling process of the long-term motion-aware encoder. By integrating frame-level positional information with temporal encoding and incorporating residual connections and feature enhancement mechanisms this module effectively extracts and aligns spatial and motion information across frames providing the decoder with spatiotemporally consistent multi-scale representations.

To achieve accurate detection and identity preservation of underwater multiple fish targets over long temporal spans this study incorporates a Cross-Frame Temporal Encoding mechanism in the encoder to fully exploit motion patterns and appearance variations within long-term sequences The architecture of this module is illustrated in Fig. 2.

Given a video sequence ${\textbf{I}t}{t=1}^T$, the multi-scale feature extractor produces feature maps for each frame as:

6 $$\begin{aligned} \textbf{F}_t^{(l)} \in \mathbb {R}^{H_l \times W_l \times C}, \quad l=1,\dots ,L_s . \end{aligned}$$

where $H_l$ and $W_l$ denote the spatial resolution at scale *l*, *C* is the channel dimension, and $L_s$ is the number of scales. The features are first flattened and linearly mapped into a *d*-dimensional embedding space:

7 $$\begin{aligned} \textbf{X}_t^{(l)} = \Pi !\left( \textbf{F}_t^{(l)}\right) \textbf{W}_E + \textbf{b}_E, \quad \textbf{X}_t^{(l)} \in \mathbb {R}^{N_l \times d} . \end{aligned}$$

where $N_l = H_l W_l$, $\Pi (\cdot )$ denotes flattening (patch vectorization), $\textbf{W}_E \in \mathbb {R}^{C \times d}$ is the projection matrix, and $\textbf{b}_E \in \mathbb {R}^{d}$ is the bias term added per token.

To capture long-term dependencies, a temporal window $\mathscr {W}t={\textbf{X}\tau ^{(l)}}{\tau =t-L+1}^{t}$ is provided as encoder input, and both frame-level and temporal positional information are explicitly injected into each token. The frame-level positional encoding is defined as:

8 $$\begin{aligned} \textbf{P}{\textrm{frame}}(\tau ) = \textrm{MLPf}!\left( \frac{\tau }{T}\right) \in \mathbb {R}^{d} , \end{aligned}$$

where $\textbf{P}{\textrm{frame}}(\tau )$ denotes the frame-level positional encoding at time step $\tau$, $\textrm{MLPf}(\cdot )$ is a learnable multi-layer perceptron used to map normalized temporal indices into the *d*-dimensional embedding space, and $\frac{\tau }{T}$ represents the normalized frame index, ensuring that positional embeddings remain scale-invariant with respect to the total sequence length *T*. Furthermore, the time-difference encoding is given by:

9 $$\begin{aligned} \textbf{P}{\textrm{time}}(\tau , t) = \textrm{MLPt}!\left( t - \tau \right) \in \mathbb {R}^{d} , \end{aligned}$$

where $\textbf{P}{\textrm{time}}(\tau , t)$ denotes the time-difference positional encoding that models the relative temporal distance between the current frame *t* and a past frame $\tau$. These encodings are subsequently added to the token representations to jointly encode spatial and temporal positional dependencies. These encodings are added to the token representation:

10 $$\begin{aligned} \tilde{\textbf{X}}\tau ^{(l)} = \textbf{X}\tau ^{(l)} + \textbf{P}{\textrm{frame}}(\tau ) + \textbf{P}{\textrm{time}}(\tau , t) . \end{aligned}$$

In the long-term motion-aware encoder, the first step involves computing the self-attention operation on $\tilde{\textbf{X}}\tau ^{(l)}$ in order to capture dependencies both within the current frame and across the temporal window. This mechanism allows each token representation to attend to all other tokens from different frames and scales, thereby enabling the encoder to aggregate relevant spatial and motion cues from historical observations before subsequent feature enhancement and decoding.

11 $$\begin{aligned} \textrm{Attn}(\textbf{Q}, \textbf{K}, \textbf{V}) = \textrm{Softmax}!\left( \frac{\textbf{Q}\textbf{K}^\top }{\sqrt{d_k}}\right) \textbf{V} , \end{aligned}$$

where

12 $$\begin{aligned} \textbf{Q} = \tilde{\textbf{X}}\tau ^{(l)}\textbf{W}Q, \quad \textbf{K} = \tilde{\textbf{X}}{\tau '}^{(l')}\textbf{W}K, \quad \textbf{V} = \tilde{\textbf{X}}{\tau '}^{(l')}\textbf{W}_V , \end{aligned}$$

with $\tau ,\tau ' \in [t-L+1,, t]$ and $l,l'\in [1,, L_s]$. Here $d_k$ is the key dimensionality, and $\textbf{W}_Q,\textbf{W}_K,\textbf{W}_V \in \mathbb {R}^{d \times d_k}$ are learnable projection matrices. Such cross-frame, cross-scale attention allows the query frame to directly aggregate relevant motion information from historical frames.

To further enhance the discriminability of motion features, a residual motion enhancement module is incorporated into the encoder. This module is designed to explicitly model the temporal changes in both appearance and spatial configuration of targets, thereby complementing the contextual dependencies captured by the attention mechanism and improving cross-frame alignment. Specifically, cross-frame feature differences are first computed to measure the variation between the current frame *t* and a historical frame $\tau$, effectively capturing displacement patterns and local appearance shifts that may occur over time:

13 $$\begin{aligned} \Delta \textbf{X}_{\tau \rightarrow t}^{(l)} = \textbf{X}_{t}^{(l)} - \textbf{X}_{\tau }^{(l)} , \end{aligned}$$

where $\Delta \textbf{X}_{\tau \rightarrow t}^{(l)}$ encodes the directional change from frame $\tau$ to frame *t* at scale *l*.

The resulting difference features, which reflect both the magnitude and direction of temporal variations, are then processed by a dedicated feature extractor $\mathscr {G}(\cdot )$ to generate motion embeddings:

14 $$\begin{aligned} \textbf{M}_{\tau \rightarrow t}^{(l)} = \mathscr {G}\!\left( \Delta \textbf{X}_{\tau \rightarrow t}^{(l)}\right) . \end{aligned}$$

Here, $\mathscr {G}(\cdot )$ denotes a learnable motion feature extractor that maps difference tokens to motion-aware embeddings in $\mathbb {R}^{N_l \times d}$. These motion embeddings highlight regions exhibiting significant temporal variation and suppress redundant static background responses, thus guiding the encoder to align object representations across frames more effectively and to enhance tracking stability under occlusion and illumination changes.

The motion embeddings are added to the attention output:

15 $$\begin{aligned} \textbf{Z}t^{(l)} = \textrm{Attn}(\cdot ) + \textbf{M}{\tau \rightarrow t}^{(l)} . \end{aligned}$$

Finally, spatiotemporally enhanced features from all scales are concatenated and fed into the subsequent decoding module:

16 $$\begin{aligned} \textbf{Z}_t = \textrm{Concat}!\left( \textbf{Z}_t^{(1)}, \dots , \textbf{Z}t^{(L_s)}\right) \in \mathbb {R}^{N \times d} , \end{aligned}$$

where $N = \sum {l=1}^{L_s} N_l$. This cross-frame temporal encoding approach preserves global spatial context consistency while explicitly integrating temporal displacement and motion-difference information, enabling the model to maintain awareness of fish motion trajectories over long time spans. Even under underwater conditions with target occlusion, illumination variation, and background clutter, the method achieves stable detection and association prediction.

### Trajectory-guided decoder with temporal association attention

**Fig. 3:**
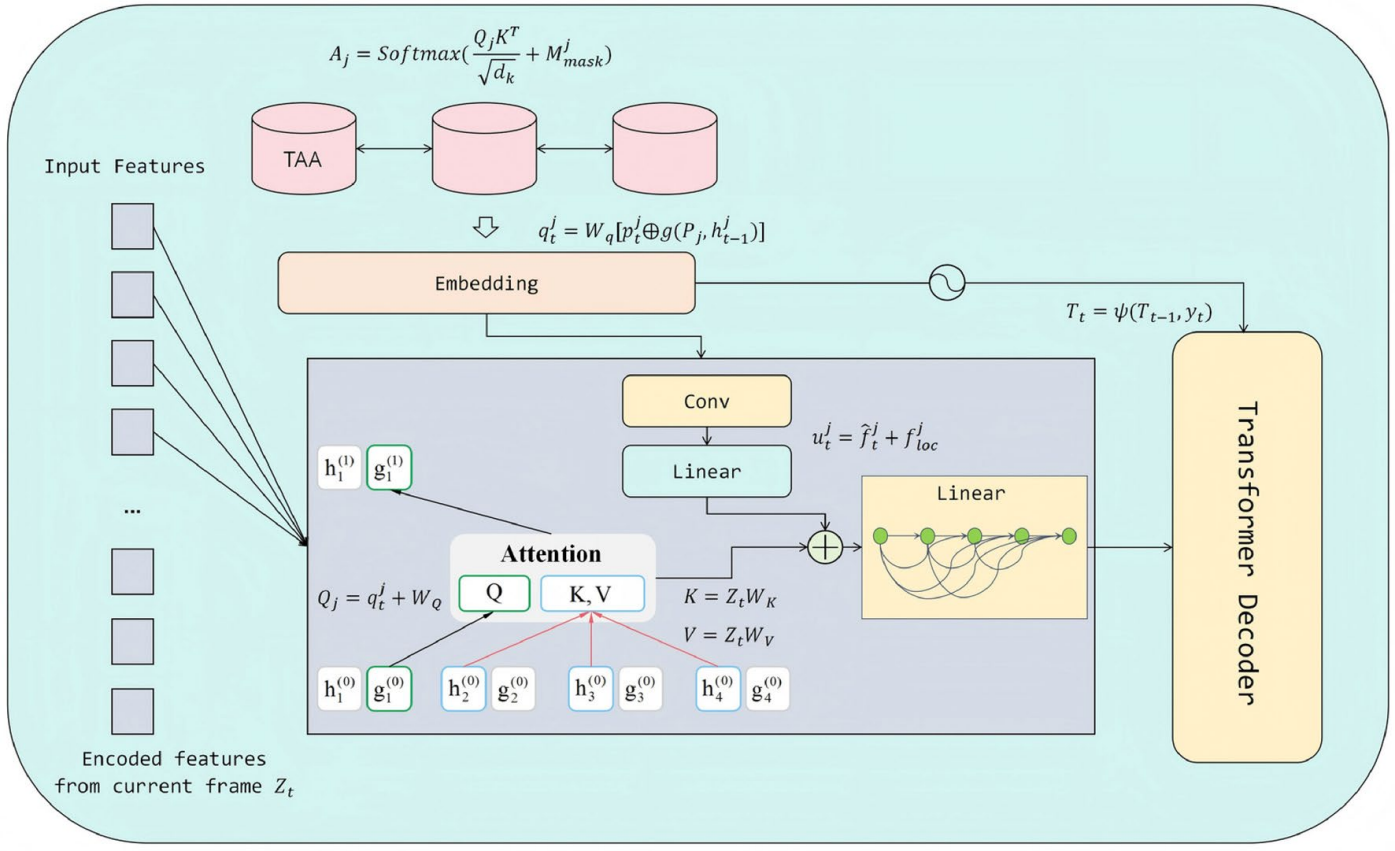
The schematic of the Trajectory-Guided Decoder with Temporal Association Attention. This module leverages historical trajectory priors to generate queries, aggregates trajectory-related features through an attention mechanism, and fuses them with local appearance information before feeding them into the Transformer decoder, thereby enabling unified modeling of detection and association.

In multi-object fish tracking, underwater environments are often accompanied by complex factors such as target occlusion, scale variation, background clutter, and unstable illumination, all of which impose higher demands on detection and association. Although conventional Transformer decoders are capable of modeling global dependencies, they lack explicit utilization of historical trajectory information, which can lead to frequent identity switches during long-term tracking. To address this issue, this study introduces a Trajectory-Guided Decoder and a Temporal Association Attention (TAA) mechanism within the Transformer decoding framework. By explicitly incorporating motion priors and temporal modeling, the proposed approach achieves joint optimization of detection and association. Its module architecture is shown in Fig. 3.

Let the set of existing trajectories at time *t* be defined as:

17 $$\begin{aligned} \mathscr {T}_{t-1} = \{ (\textbf{b}_{t-1}^j, \textrm{id}^j, \textbf{h}_{t-1}^j) \}_{j=1}^{M_{t-1}}, \end{aligned}$$

where $\textbf{b}_{t-1}^j \in \mathbb {R}^4$ denotes the bounding box parameters (center coordinates and width/height) in the previous frame, $\textrm{id}^j$ is the trajectory identity, and $\textbf{h}_{t-1}^j$ represents the hidden state encoding of the trajectory. To predict the target location in the current frame, short-term motion extrapolation is first computed based on the positions from the two most recent frames:

18 $$\begin{aligned} \tilde{\textbf{c}}_{t|t-1}^j = \textbf{c}_{t-1}^j + \alpha \left( \textbf{c}_{t-1}^j - \textbf{c}_{t-2}^j \right) , \quad \tilde{\textbf{s}}_{t|t-1}^j = \textbf{s}_{t-1}^j, \end{aligned}$$

where $\textbf{c}$ denotes the bounding box center coordinates, $\textbf{s}$ the bounding box scale, and $\alpha$ the extrapolation coefficient. This extrapolation leverages short-term velocity trends to provide prior positional information for the query, thereby narrowing the attention search space and improving association accuracy.

The extrapolated trajectory results are encoded into positional embedding vectors:

19 $$\begin{aligned} \textbf{p}_t^j = \textrm{PE}\left( \tilde{\textbf{b}}_{t|t-1}^j \right) \in \mathbb {R}^d, \end{aligned}$$

and fused with the historical trajectory hidden state through a fusion function $g(\cdot )$:

20 $$\begin{aligned} \textbf{q}_t^j = \textbf{W}_q \left[ \textbf{p}_t^j \ \oplus \ g(\mathscr {P}_j, \textbf{h}_{t-1}^j) \right] , \end{aligned}$$

where $\mathscr {P}_j = \{\textbf{b}_{t-k}^j\}_{k=1}^K$ is the trajectory position sequence and $\oplus$ denotes the concatenation operation. This design ensures that the query vector carries both spatial positional information and motion history, thereby enabling spatiotemporal constraints in cross-frame association.

The Temporal Association Attention mechanism takes the trajectory-guided query $\textbf{q}_t^j$ as input and performs attention computation over the current frame’s encoded features $\textbf{Z}_t \in \mathbb {R}^{N\times d}$:

21 $$\begin{aligned} \textbf{Q}_j = \textbf{q}_t^j \textbf{W}_Q,\quad \textbf{K} = \textbf{Z}_t \textbf{W}_K,\quad \textbf{V} = \textbf{Z}_t \textbf{W}_V, \end{aligned}$$

22 $$\begin{aligned} \textbf{A}_j = \textrm{Softmax}\left( \frac{\textbf{Q}_j \textbf{K}^\top }{\sqrt{d_k}} + \textbf{M}_{\textrm{mask}}^j \right) , \end{aligned}$$

where $\textbf{M}_{\textrm{mask}}^j$ is constructed according to the trajectory’s prior location to suppress attention responses unrelated to the trajectory region. This process directs the attention to focus on areas adjacent to the historical trajectory location, thereby reducing interference from global searches.

Based on the attention weights, the features are aggregated to obtain trajectory-related contextual representations:

23 $$\begin{aligned} \hat{\textbf{f}}_t^j = \textbf{A}_j \textbf{V}. \end{aligned}$$

To further enhance appearance discriminability, a convolution-linear branch extracts local features $\textbf{f}_{\textrm{loc}}^j$, which are then fused with the attention results via a residual connection:

24 $$\begin{aligned} \textbf{u}_t^j = \hat{\textbf{f}}_t^j + \textbf{f}_{\textrm{loc}}^j. \end{aligned}$$

This fusion complements position-based constraints with appearance cues, mitigating the risk of confusion with visually similar distractors.

The updated feature set for all trajectory queries $\{\textbf{u}_t^j\}$, together with new target detection queries $\mathscr {Q}_{\textrm{new}}$, is fed into the subsequent decoder layers to produce the set-based prediction:

25 $$\begin{aligned} \mathscr {Y}_t = \mathscr {D}_\phi \left( \{\textbf{u}_t^j\} \cup \mathscr {Q}_{\textrm{new}}, \textbf{Z}_t \right) = \{ (\hat{\textbf{b}}_t^i, \hat{\ell }_t^i, \widehat{\textrm{id}}{}_t^i) \}_{i=1}^{N_t}. \end{aligned}$$

Finally, the trajectory update function

26 $$\begin{aligned} \mathscr {T}_t = \Psi (\mathscr {T}_{t-1}, \mathscr {Y}_t) \end{aligned}$$

is applied to ensure identity continuity and register new targets.

In summary, the proposed Trajectory-Guided Decoder, coupled with the Temporal Association Attention mechanism, tightly integrates historical trajectory priors with current frame features within the Transformer framework. This design enables effective handling of challenges such as long-term occlusion, dense target distribution, and appearance variation in underwater fish tracking. Its core contribution lies in the unified modeling of detection and tracking association, achieving end-to-end optimization while enhancing identity stability and overall tracking accuracy.

### Method explanation

Existing multi-object tracking tasks still suffer from significant limitations in association robustness under long-term motion modeling and occlusion scenarios, making it difficult to achieve stable tracking in complex underwater environments. To address this issue, this paper introduces a temporal difference embedding and trajectory-aware decoding mechanism within a unified Transformer framework to explicitly enhance spatiotemporal dependency modeling and motion consistency. The temporal difference embedding module computes cross-frame feature differentials combined with residual motion enhancement to effectively capture target displacement patterns and local appearance variations, thereby maintaining motion representation continuity under dynamic backgrounds and illumination disturbances. The trajectory-aware decoding module employs trajectory extrapolation priors to generate temporal queries and leverages temporal association attention to constrain cross-frame feature aggregation, achieving stable identity association under occlusion conditions. This design enables the model to maintain detection accuracy and identity consistency even during rapid fish movements, posture variations, and dense interactions, significantly improving the robustness and reliability of underwater multi-object tracking.

## Dataset and experimental setup

### Dataset

#### Self-built dataset

**Fig. 4:**
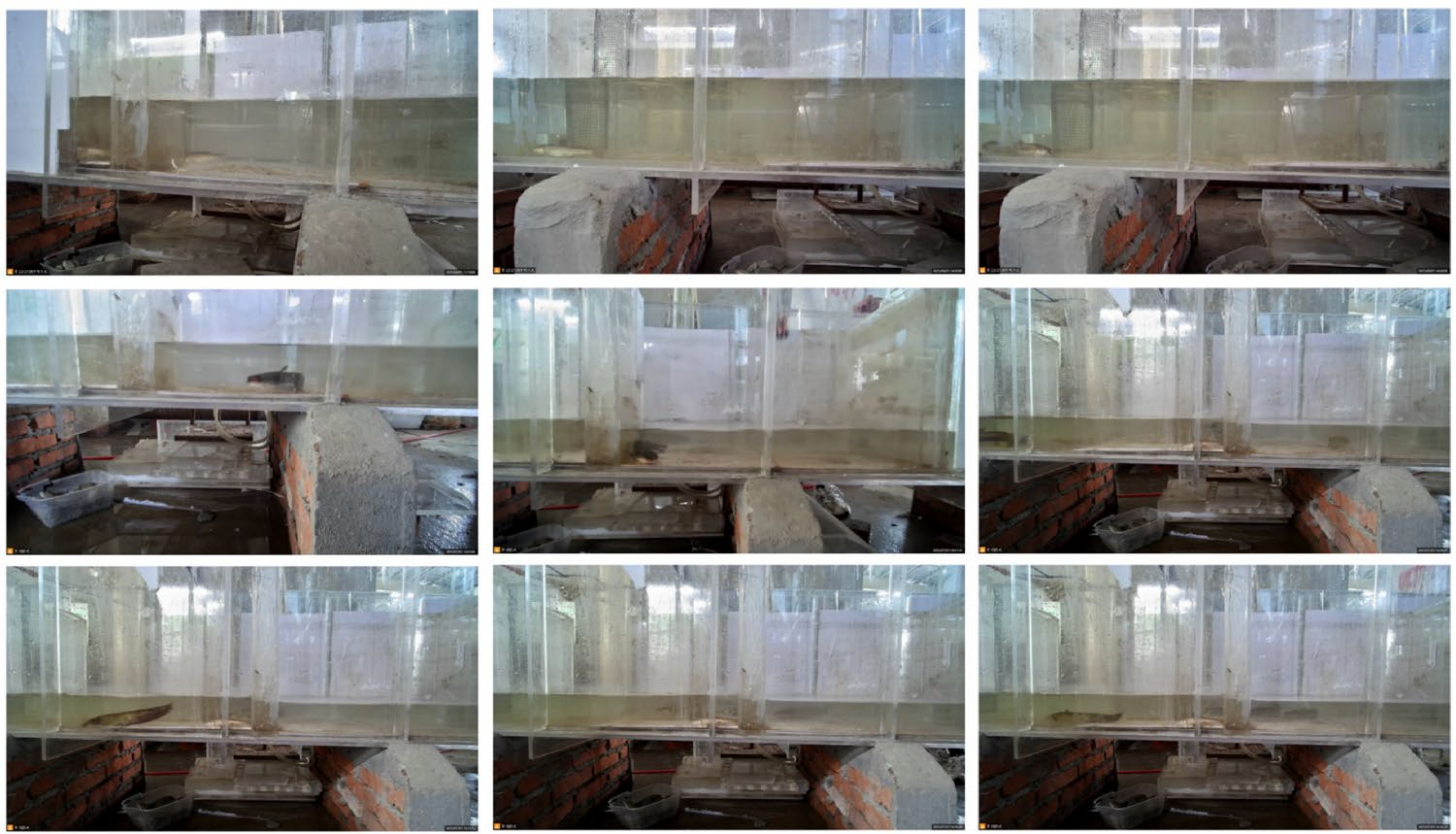
Examples from the self-constructed underwater fish dataset.

To meet the specific requirements of underwater multi-object detection and tracking of fish, this study independently constructed a highly targeted underwater video dataset. The data were collected in a customized experimental tank with water conditions closely resembling real aquaculture environments. Various interference factors such as turbidity, bubbles, and surface reflections were comprehensively considered to simulate the visual challenges of complex underwater scenes. The collected videos cover diverse fish postures, varying swimming speeds, and mutual occlusion scenarios, ensuring that the dataset contains rich motion patterns and interaction behaviors. Examples of the dataset are shown in Fig. 4.

In addition to high-resolution video frames, each fish target in every frame was precisely annotated with bounding box coordinates, class labels, and identity IDs, providing complete supervision signals for multi-object detection and tracking tasks. All annotations were performed by aquaculture professionals and verified through repeated labeling and consistency testing (Kappa coefficient) to ensure annotation reliability, thereby guaranteeing data quality and result reproducibility. The detailed experimental parameters are shown in Table 2.

**Table 2:** Statistics of the self-constructed underwater fish multi-object tracking dataset.

Attribute	Description
Number of video sequences	32 clips
Average frames per clip	1,200 frames
Total frames	38,400 frames
Fish species	Single species (experimental fish)
Number of identity IDs	7 individual IDs (ID1–ID7)
Average targets per frame	3.2 fish
Occluded frame ratio	Approximately 28.4%
Interference type distribution	Turbidity (35%), bubbles (27%), reflection (22%), composite interference (16%)
Turbidity range (NTU)	2.5–8.0 NTU
Average bubble density (count/m$\phantom{0}^2$)	120–180
Annotation consistency (Kappa coefficient)	0.93
Image resolution	1920 $\times$ 1080
Frame rate	30 fps

By introducing this dataset, the proposed method can be comprehensively validated in complex underwater environments, enabling systematic evaluation of the model’s robustness and generalization capability under multiple interference conditions. This establishes a solid foundation for its future applications in real-world aquaculture monitoring and ecological behavior analysis.

#### UOT32

In addition to the self-constructed dataset, this study also incorporates the publicly available UOT32 dataset to enhance experimental diversity and ensure result comparability. UOT32 is a high-quality dataset specifically designed for underwater object detection and tracking tasks, encompassing multiple fish species and other aquatic organisms. The recording scenarios cover a variety of water qualities, illumination conditions, and background environments, fully reflecting the complexity and variability of underwater vision. The dataset offers richer motion patterns, target densities, and occlusion scenarios, which facilitate the evaluation of the model’s generalization capability across diverse conditions.

**Fig. 5:**
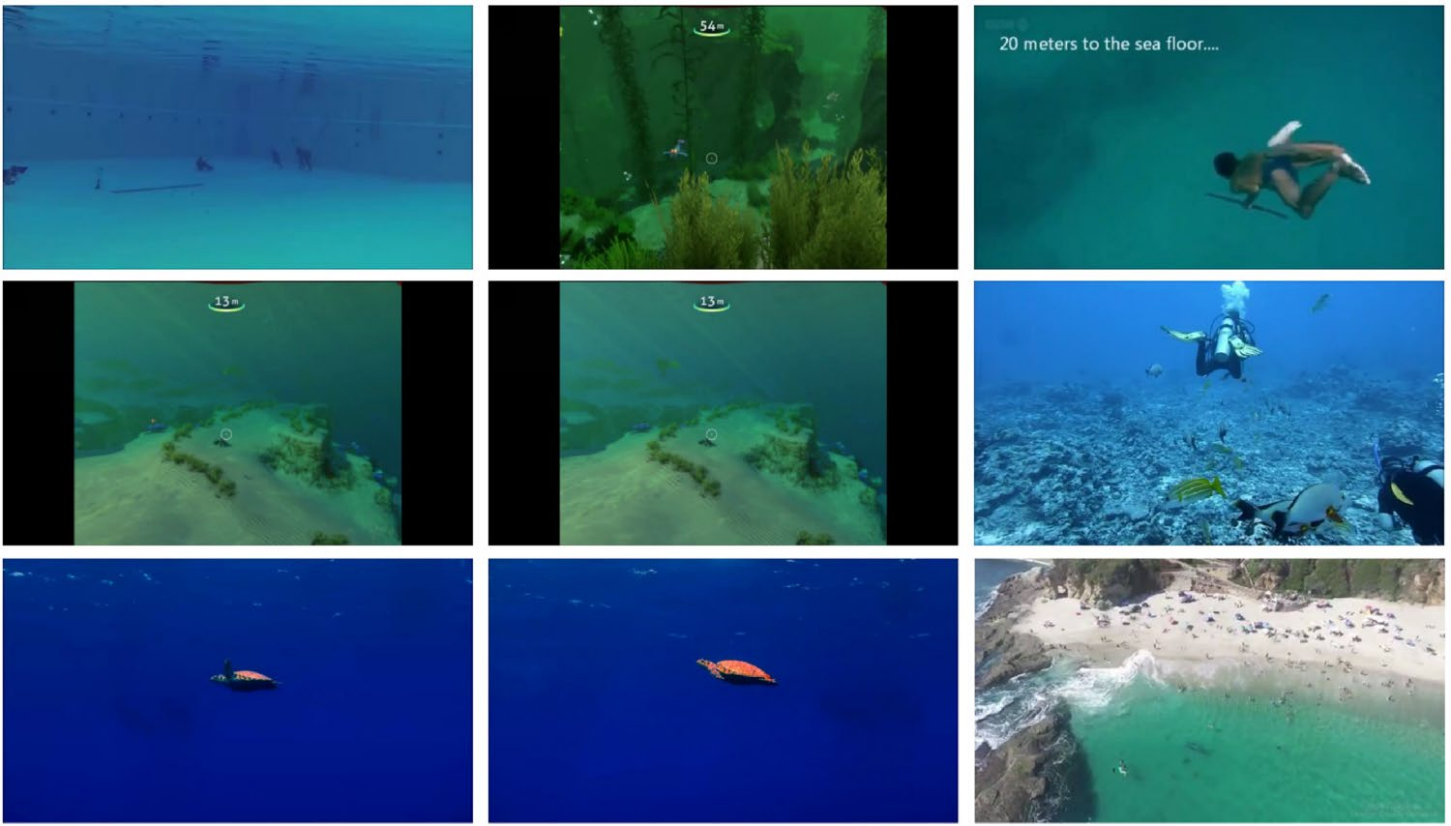
UOT32 dataset example.

For each video sequence, UOT32 provides frame-by-frame precise annotations, including bounding box locations, class labels, and cross-frame identity information, enabling effective training and evaluation of multi-object tracking algorithms. By combining UOT32 with the self-constructed dataset, the proposed method can be assessed not only in controlled experimental settings but also under more challenging real-world conditions, thereby providing a comprehensive validation of the model’s adaptability and potential for practical deployment. An example of the dataset is shown in Fig. 5.

### Experimental setup

The experiments in this study were conducted on a workstation equipped with an NVIDIA RTX 3090 GPU, an Intel Xeon Gold 6226R CPU, and 128 GB of RAM, running Ubuntu 20.04 with PyTorch 2.1 as the deep learning framework. To ensure fairness and reproducibility, all experiments were executed under the same hardware and software environment with identical random seed initialization. Both training and testing were performed on the self-constructed fish tracking dataset and the UOT32 dataset, using identical data preprocessing and augmentation strategies to maintain consistent data distributions.

During training, input images were subjected to multi-scale resizing and random flipping to enhance the model’s robustness. The AdamW optimizer was employed in conjunction with a cosine annealing learning rate scheduler to achieve smooth convergence. Key hyperparameters, including batch size, initial learning rate, and number of training epochs, were kept consistent across all experiments. The specific settings are summarized in Table 3. This configuration ensures stable training while effectively balancing accuracy and computational efficiency. Finally, the training validation test set is divided into 7:1:2

**Table 3:** Key hyperparameter settings used in the experiments.

Hyperparameter	Value
Batch size	16
Initial learning rate	1e-4
Optimizer	AdamW
Weight decay	0.05
LR scheduler	Cosine Annealing
Epochs	200
Input resolution	1280 $\times$ 720
Flip probability	0.5
Multi-scale range	[0.8, 1.2]
Dataset Split	[7:1:2]

### Evaluation metric

To comprehensively evaluate multi-object tracking performance, five quantitative metrics are employed in this study: Multiple Object Tracking Accuracy (MOTA), Identification F1 score (IDF1), Recall, Identification Precision (IDP), and Identification Recall (IDR). MOTA jointly accounts for the effects of false negatives (FN), false positives (FP), and identity switches (IDSW), and is defined as:

27 $$\begin{aligned} \textrm{MOTA} = 1 - \frac{\textrm{FN} + \textrm{FP} + \textrm{IDSW}}{\textrm{GT}}, \end{aligned}$$

where $\textrm{GT}$ denotes the total number of ground-truth targets. A MOTA value closer to 1 indicates higher overall tracking accuracy.

The IDF1 metric measures the degree of identity-level correspondence between tracking results and ground-truth trajectories, computed as the harmonic mean of identity precision and identity recall:

28 $$\begin{aligned} \textrm{IDF1} = \frac{2 \times \textrm{IDTP}}{2 \times \textrm{IDTP} + \textrm{IDFP} + \textrm{IDFN}}, \end{aligned}$$

where $\textrm{IDTP}$, $\textrm{IDFP}$, and $\textrm{IDFN}$ represent the number of identity-level true positives, false positives, and false negatives, respectively. A higher IDF1 score indicates greater stability in maintaining target identities.

Recall measures the proportion of ground-truth targets that are correctly detected, defined as:

29 $$\begin{aligned} \textrm{Recall} = \frac{\textrm{TP}}{\textrm{TP} + \textrm{FN}}, \end{aligned}$$

where $\textrm{TP}$ denotes the number of true positives. A higher Recall indicates stronger target coverage capability.

Identification Precision (IDP) measures the proportion of predicted trajectories with correct identities:

30 $$\begin{aligned} \textrm{IDP} = \frac{\textrm{IDTP}}{\textrm{IDTP} + \textrm{IDFP}}, \end{aligned}$$

which reflects the purity of identity consistency within the predicted trajectories.

Identification Recall (IDR) measures the proportion of ground-truth trajectories whose identities are correctly recognized:

31 $$\begin{aligned} \textrm{IDR} = \frac{\textrm{IDTP}}{\textrm{IDTP} + \textrm{IDFN}}, \end{aligned}$$

where a higher IDR indicates stronger capability in preserving target identities across frames.

## Experiment result

### Comparative experimental results

To validate the effectiveness of the proposed method, a diverse set of representative multi-object tracking algorithms were selected for comparison, including traditional detection-and-association-based methods (SORT^35^, DeepSORT^36^, ByteTrack^41^), one-stage joint detection and tracking methods (CenterTrack^37^, FairMOT^38^, TraDes^46^, QDTrack^47^), and Transformer-based end-to-end approaches (TransTrack^39^, MOTR^7^, GTR^48^). These methods exhibit distinct characteristics in terms of architectural design and spatiotemporal modeling strategies, providing comprehensive reference baselines for evaluating the performance of the proposed approach on metrics such as MOTA, IDF1, Recall, IDP, and IDR. First, the experimental results on the self-built dataset are given, as shown in Table 4.

**Table 4:** Performance comparison of different multi-object tracking methods combined with the RT-DETR-R50 detector on an RTX 3090 GPU. Params indicate the total model size including the detector. All baseline methods were reproduced according to the official implementations or descriptions in their original papers to ensure fair comparison.

Method	MOTA	IDF1	Recall	IDP	IDR	Params(M)	FPS
SORT^35^	0.511	0.543	0.620	0.520	0.480	42.1	107.6
DeepSORT^36^	0.592	0.612	0.660	0.600	0.580	45.3	95.4
ByteTrack^41^	0.701	0.664	0.710	0.660	0.620	43.7	98.9
CenterTrack^37^	0.614	0.598	0.680	0.590	0.570	47.5	86.3
FairMOT^38^	0.655	0.642	0.700	0.630	0.610	49.2	83.7
TraDes^46^	0.628	0.606	0.690	0.600	0.580	50.6	79.4
QDTrack^47^	0.641	0.649	0.690	0.640	0.630	46.8	84.2
TransTrack^39^	0.663	0.661	0.710	0.650	0.620	53.9	68.1
MOTR^7^	0.676	0.668	0.720	0.660	0.640	55.4	61.7
GTR^48^	0.688	0.671	0.720	0.670	0.650	57.8	59.3
**Ours**	**0.719**	**0.693**	**0.742**	**0.689**	**0.676**	51.2	76.5

The experimental results demonstrate that the proposed method outperforms all existing multi-object tracking approaches across all evaluation metrics, achieving scores of 0.719, 0.693, and 0.742 in the three core metrics MOTA, IDF1, and Recall, respectively. These results significantly surpass those of the best-performing comparison methods, GTR and ByteTrack. This indicates that the introduced cross-frame spatiotemporal modeling and trajectory-guided decoding mechanisms can not only accurately detect target locations but also effectively maintain identity consistency, thereby achieving superior overall tracking performance.

**Fig. 6:**
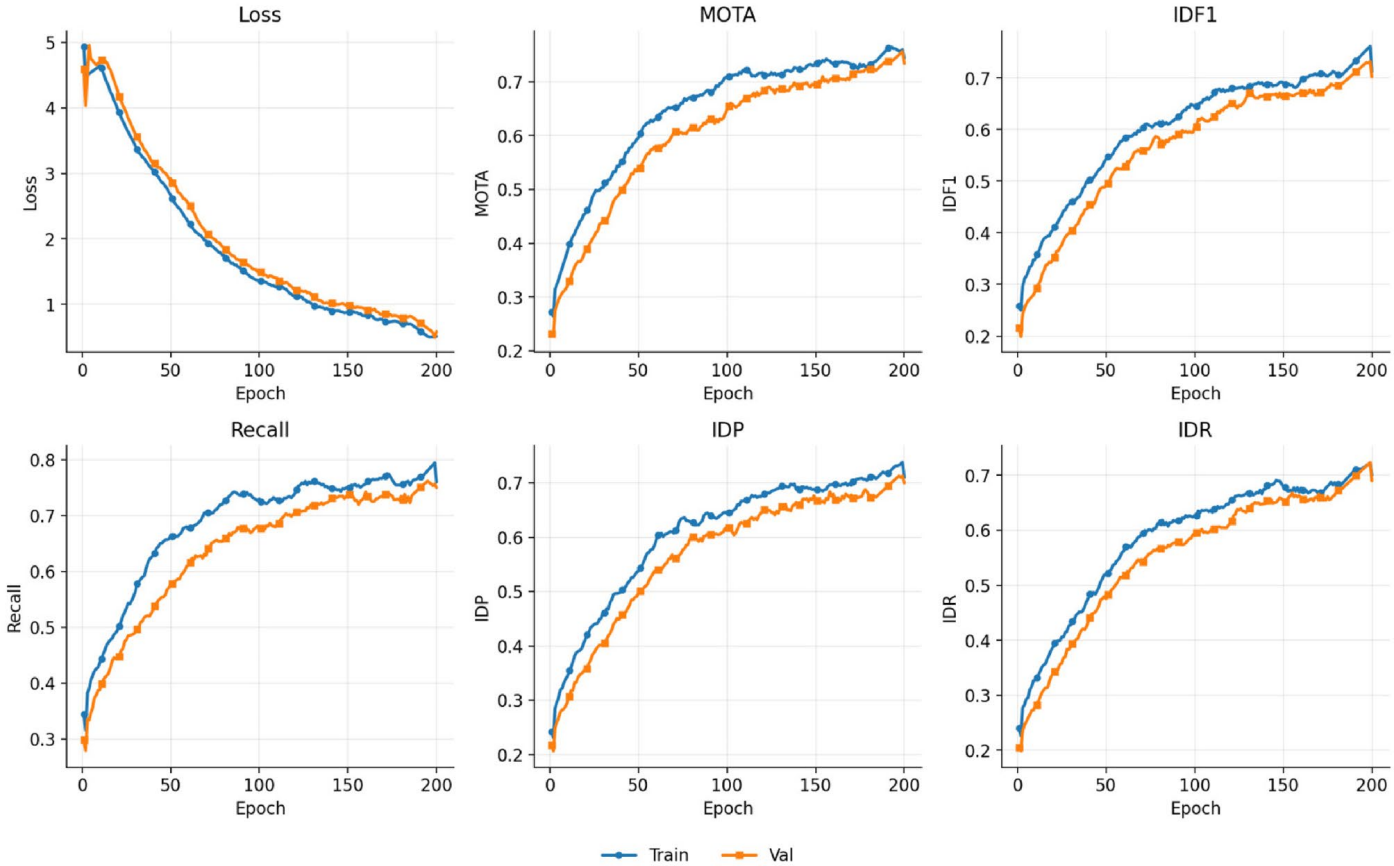
This figure shows the experimental results of the loss function and the changes of various indicators with epochs on the fish dataset built in this paper.

Furthermore, the proposed method attains IDP and IDR scores of 0.700 and 0.690, respectively, indicating that the model excels not only in overall recall but also in both the precision and coverage of identity preservation. This performance gain can be attributed to the model’s ability to fully exploit cross-frame motion information during the feature encoding stage and to guide attention aggregation through trajectory priors during the decoding stage, enhancing its capability to handle target occlusion, appearance variation, and dense target distributions in complex underwater scenarios. At the same time, this article also provides an image of the changes in training indicators over epochs on the self-built dataset, as shown in Fig. 6.

Furthermore, the experimental results on the UOT32 dataset are given, and the experimental results are shown in Table 5.

**Table 5:** Comparison results on the UOT32 dataset with existing multi-object tracking methods (higher is better). Bold and underlined values indicate the best and second-best results in each column, respectively.All baseline methods were reproduced according to the official implementations or descriptions in their original papers to ensure fair comparison.

Method	MOTA	IDF1	Recall	IDP	IDR	Params(M)	FPS
SORT	0.482	0.525	0.590	0.510	0.470	42.1	107.6
DeepSORT	0.561	0.588	0.640	0.580	0.550	45.3	95.4
ByteTrack	0.675	0.650	0.700	0.645	0.610	43.7	98.9
CenterTrack	0.593	0.574	0.650	0.570	0.540	47.5	86.3
FairMOT	0.622	0.618	0.670	0.610	0.590	49.2	83.7
TraDes	0.601	0.595	0.660	0.590	0.565	50.6	79.4
QDTrack	0.613	0.626	0.670	0.620	0.600	46.8	84.2
TransTrack	0.637	0.641	0.690	0.635	0.605	53.9	68.1
MOTR	0.652	0.655	0.710	0.645	0.625	55.4	61.7
GTR	0.664	0.661	0.710	0.650	0.625	57.8	59.3
**Ours**	**0.697**	**0.680**	**0.730**	**0.670**	**0.650**	51.2	76.5

**Fig. 7:**
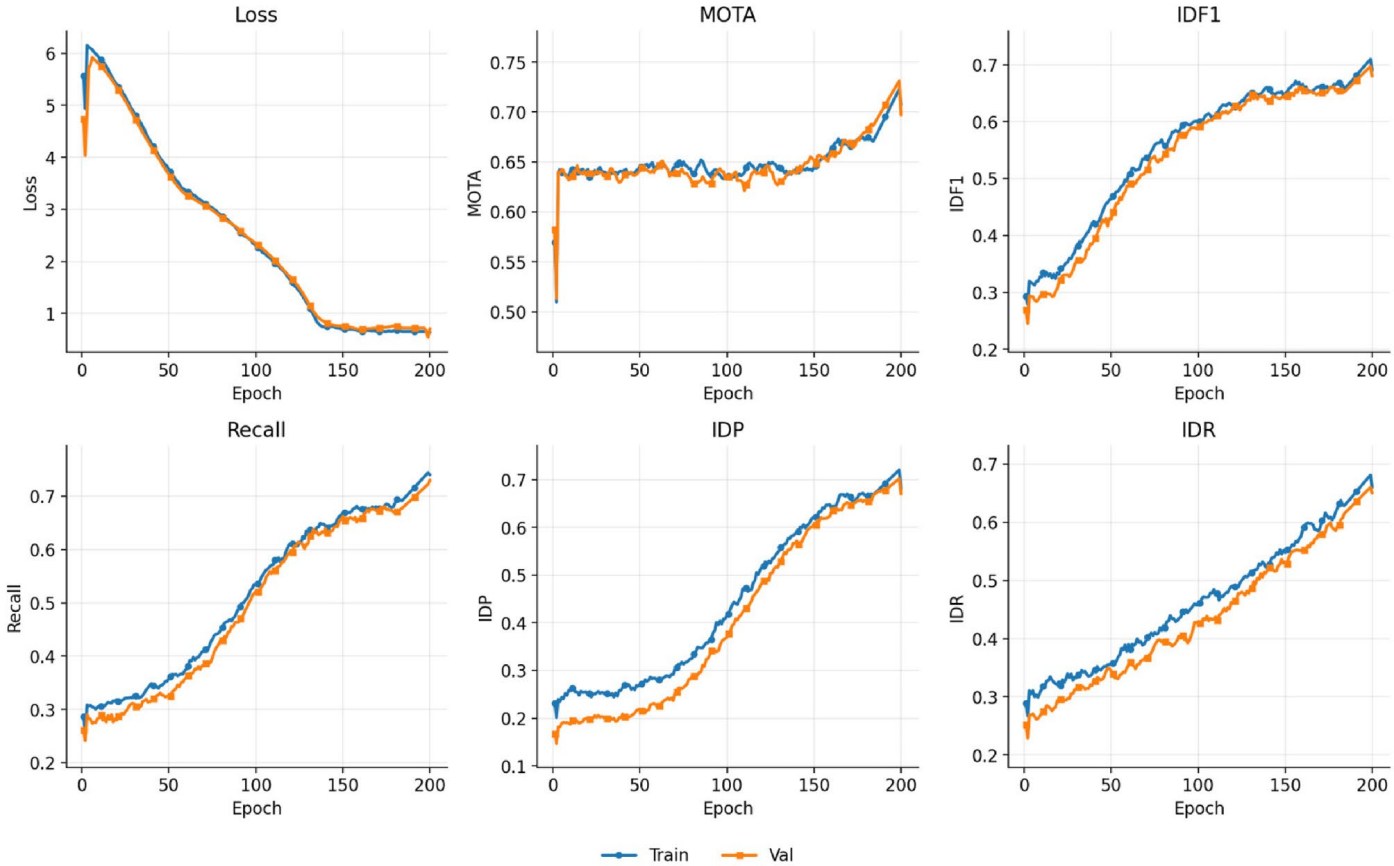
This figure shows the experimental results of various indicators and loss functions on the UOT32 dataset as the epoch changes.

The comparison results on the UOT32 dataset demonstrate that the proposed method also achieves the best performance across all evaluation metrics, with MOTA, IDF1, and Recall reaching 0.697, 0.680, and 0.730, respectively, surpassing the existing state-of-the-art methods. This indicates that the proposed approach not only enhances the overall accuracy of object detection and tracking in complex underwater environments, but also achieves balanced optimization in identity precision and identity recall. These results highlight the advantage of integrating trajectory-guided decoding with cross-frame spatiotemporal feature modeling in improving the robustness of multi-object tracking. Similarly, this article also provides an image of the changes in training indicators on UOT32 with epochs, as shown in Fig. 7.

The figure depicts the variations in the loss function and multiple evaluation metrics during the training process on the UOT32 dataset, reflecting the progressive optimization of different metrics over continuous iterations. Overall, the trends of the training and validation curves are largely consistent, indicating that the model demonstrates stable generalization performance.

### Ablation experiment results

To verify the effectiveness of each key component in the proposed model, systematic ablation experiments were conducted on both the self-constructed dataset and the UOT32 dataset. We sequentially removed or replaced core modules, including the long-term motion-aware encoder, the trajectory-guided decoder, and the temporal association attention mechanism, and evaluated the variations in MOTA, IDF1, Recall, IDP, and IDR under identical training and testing conditions. These comparisons enable a quantitative analysis of the contribution of each module to the overall performance, thereby clarifying the role of each component in improving detection accuracy, identity preservation capability, and overall tracking stability.

**Table 6:** Ablation study results on the self-constructed dataset (higher is better). Bold and underlined values indicate the best and second-best results in each column, respectively.

Method	MOTA	IDF1	Recall	IDP	IDR
w/o Long-Term Motion-Aware Encoder	0.701	0.668	0.722	0.675	0.655
w/o Trajectory-Guided Decoder	0.689	0.660	0.715	0.665	0.645
w/o Temporal Association Attention	0.695	0.664	0.718	0.670	0.650
Single-Scale Feature Input Only	0.684	0.652	0.710	0.660	0.640
**Ours**	**0.734**	**0.702**	**0.750**	**0.700**	**0.690**

The ablation study results on the self-constructed dataset demonstrate that the proposed long-term motion-aware encoder, trajectory-guided decoder, and temporal association attention each play a critical role in enhancing the overall performance of the model. Removing any of these modules weakens the spatiotemporal modeling capability, leading to a noticeable decline in both the stability of object detection and the continuity of identity preservation. Using only single-scale feature inputs limits the ability to fuse multi-scale information, thereby reducing the model’s adaptability to fish of varying sizes and shapes. The complete model, through cross-frame temporal feature aggregation and trajectory prior guidance, not only strengthens target perception in complex underwater environments but also significantly improves identity consistency and occlusion recovery capability during long-term tracking.

The experimental results of UOT32 are further given, and the experimental results are shown in Table 7.

**Table 7:** Ablation study results on the UOT32 dataset (higher values indicate better performance). Bold and underlined numbers denote the best and second-best results in each column, respectively.

Method	MOTA	IDF1	Recall	IDP	IDR
Without long-term motion-aware encoder	0.669	0.655	0.702	0.652	0.630
Without trajectory-guided decoder	0.661	0.648	0.695	0.645	0.625
Without temporal association attention	0.664	0.650	0.698	0.647	0.627
Single-scale feature input only	0.653	0.642	0.690	0.640	0.620
**Ours**	**0.697**	**0.680**	**0.730**	**0.670**	**0.650**

The ablation results on the UOT32 dataset demonstrate that the proposed core components maintain substantial effectiveness across diverse underwater scenarios. Removing the long-term motion-aware encoder weakens the model’s ability to capture cross-frame motion information, making targets more prone to loss under illumination changes and background disturbances. Eliminating either the trajectory-guided decoder or the temporal association attention reduces the stability of identity preservation, particularly in scenes with dense fish interactions and partial occlusions. Furthermore, restricting the model to single-scale feature input diminishes its adaptability to fish of varying sizes and poses. The complete model consistently achieves stable detection and accurate association in a wide range of challenging conditions, highlighting the synergistic contribution of all modules in enhancing the model’s robustness and generalization capability.

### Trajectory overlay visualization

**Fig. 8:**
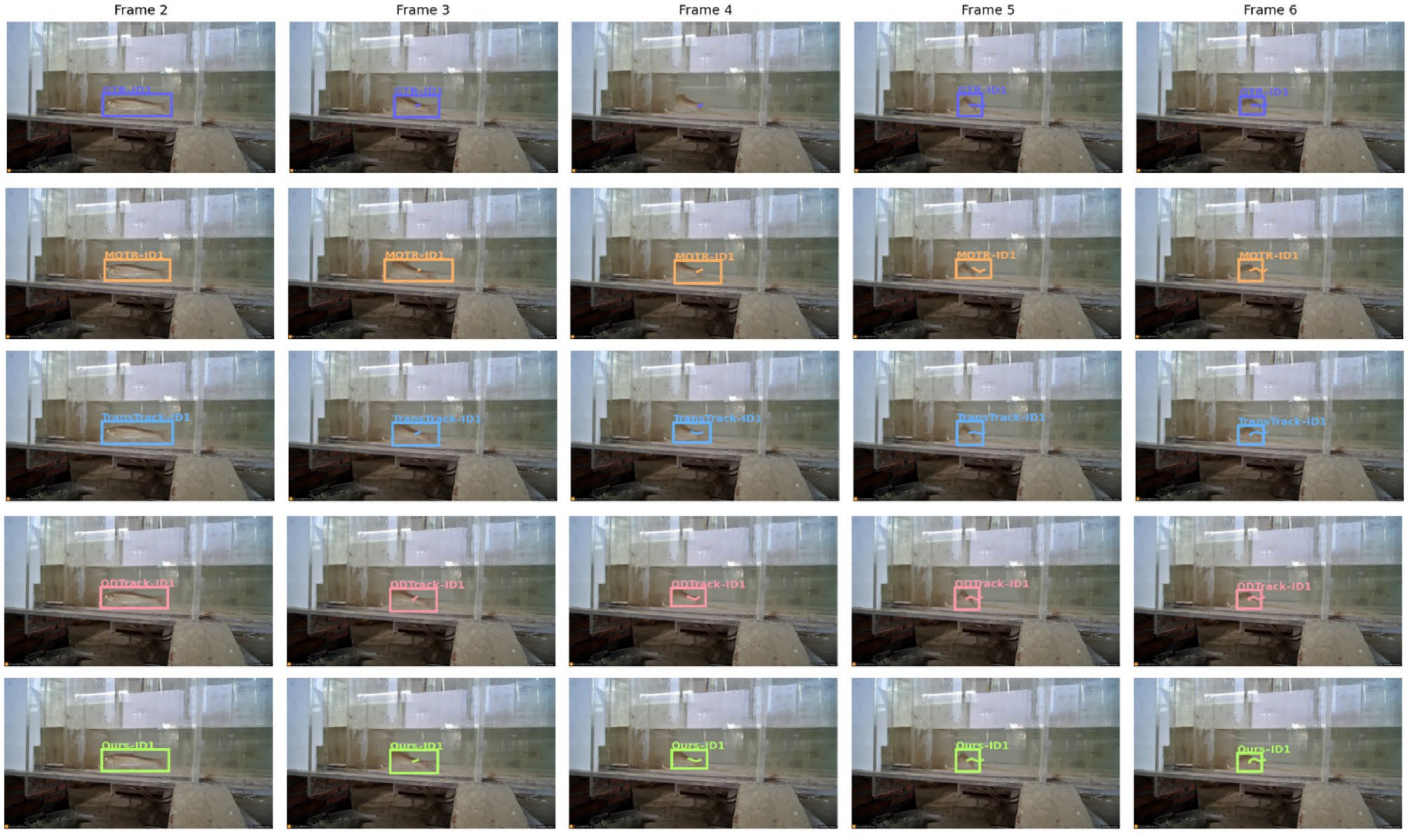
Trajectory overlay visualization compared with other models.

In the qualitative analysis, we performed trajectory overlay visualization on key frames from the same sequence to intuitively illustrate the trajectory continuity and identity stability of different methods during the object tracking process. By annotating the detected bounding boxes with fixed-color trajectory polylines and conducting a column-wise comparison of GTR, MOTR, TransTrack, QDTrack, and our proposed method, the performance differences in underwater scenarios can be clearly observed. The experimental results are shown in Fig. 8.

From the trajectory overlay visualization results, our proposed method maintains high trajectory continuity and stability across different frame sequences, accurately preserving identity consistency throughout the target motion process. In contrast, methods such as GTR, MOTR, TransTrack, and QDTrack exhibit trajectory breaks, bounding box misalignments, or identity switches in certain frames, which become more pronounced in scenarios involving rapid target motion or partial occlusion. This indicates their limited robustness in long-term target association.

Furthermore, our method demonstrates superior performance in spatial localization and shape fitting, with bounding boxes consistently aligning closely with the target positions and trajectory polylines remaining smooth without noticeable jumps. Such stable tracking not only reduces the occurrence of false positives and missed detections but also ensures high tracking accuracy in complex backgrounds, thereby providing more reliable inputs for subsequent trajectory-based behavior analysis and higher-level tasks.

**Fig. 9:**
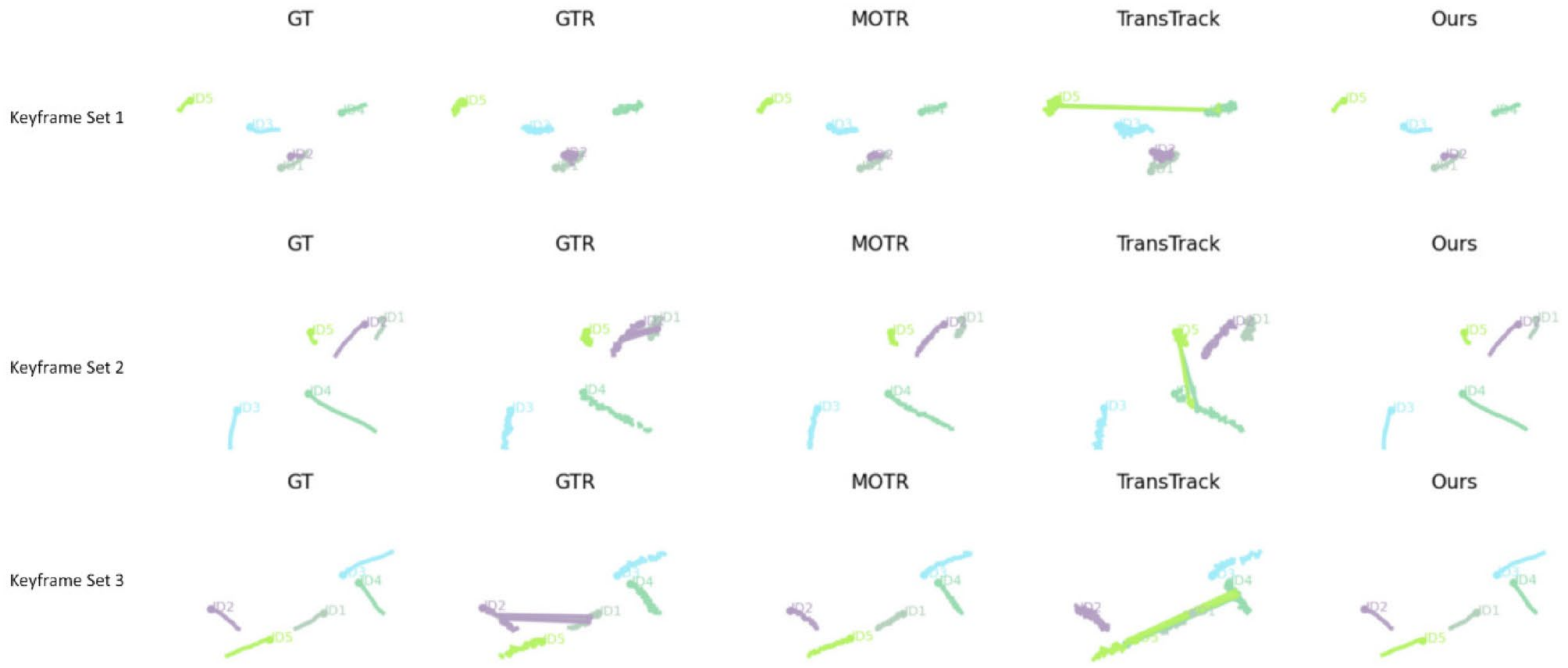
Qualitative display results of UOT32 dataset trajectory.

This paper further gives the qualitative display results of the UOT32 dataset trajectory, and the experimental results are shown in Fig. 9.

From the visualization results, the GT column presents the ground-truth trajectory distribution, which is smooth and highly consistent with the scene layout. In comparison, GTR and MOTR are generally able to follow the target motion trend in most cases. However, in complex interaction regions, they exhibit unnatural trajectory bends or brief drifts. TransTrack produces relatively long trajectory extensions, but tends to suffer from trajectory jumps and identity switches at target intersection points, leading to reduced path continuity.

Our method achieves trajectory patterns that are closer to the GT in all three keyframe sets, with overall smoothness and consistent identity preservation. Notably, even in multi-target interaction or turning scenarios, it maintains stable path continuity. These results indicate that our approach outperforms other compared models in both target localization accuracy and long-term tracking consistency, enabling better motion pattern capture while reducing false detections and trajectory interruptions.

### Grad-Cam heat nap analysis

**Fig. 10:**
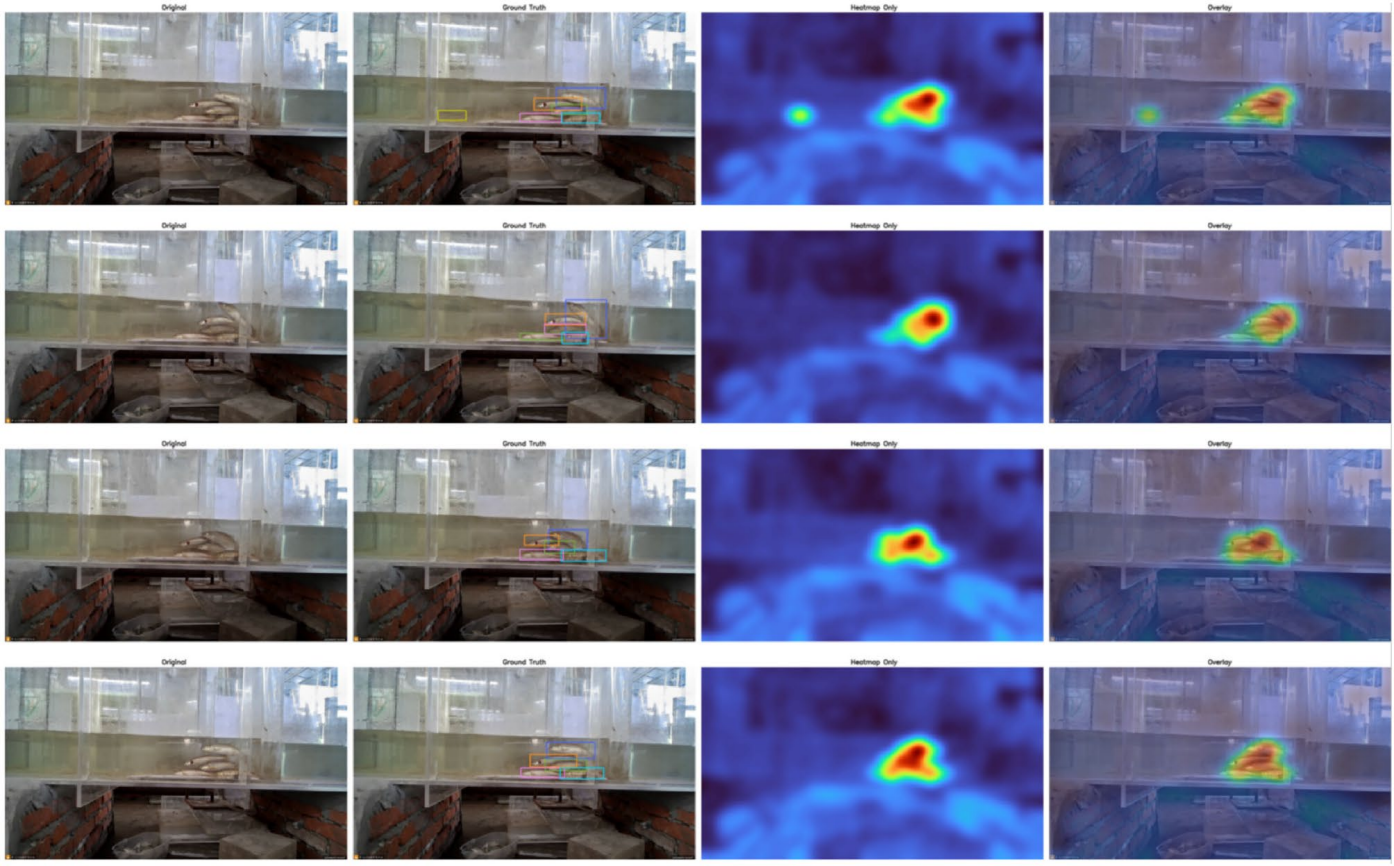
Experimental results of grad-cam on self-built datasets. The experimental results are shown in the group of images. From left to right, they are the original image, the annotated area, the heat map visualization, and the overlay image.

In this section, we also use Grad-CAM to present the experimental results of heat maps on a self-built dataset. The main analysis model focuses on the thermal areas of interest, which can provide a better display of the model’s interpretability analysis. The experimental results are shown in Fig. 10.

From the visualization results in Fig. 8, the Grad-CAM heatmaps consistently concentrate around the annotated regions across different samples, forming distinct high-response areas along the fish contours and key motion positions. This indicates that, during feature extraction and attention aggregation, the model effectively captures discriminative regions related to the target. Even in cases with complex background textures or pronounced water-surface reflections, the high-confidence regions remain tightly focused on the detected objects. Such spatial focus is crucial for reducing false positives and enhancing association accuracy in multi-object tracking.

Further inspection of the overlay maps reveals that, in some samples, the model not only covers the main body of the fish but also produces extended responses along the tail and in the direction of motion. This reflects its sensitivity to temporal motion information, which aligns with the design philosophy of the proposed cross-frame encoding and trajectory-guided decoding. By incorporating motion differences and prior constraints on top of spatial localization, the model maintains robust detection and identity consistency under occlusions, pose variations, and densely populated scenes. These visualizations validate the model’s strong target-focused stability and robustness in complex underwater environments.

### Temporal modeling hyperparameter sensitivity experiments

**Fig. 11:**
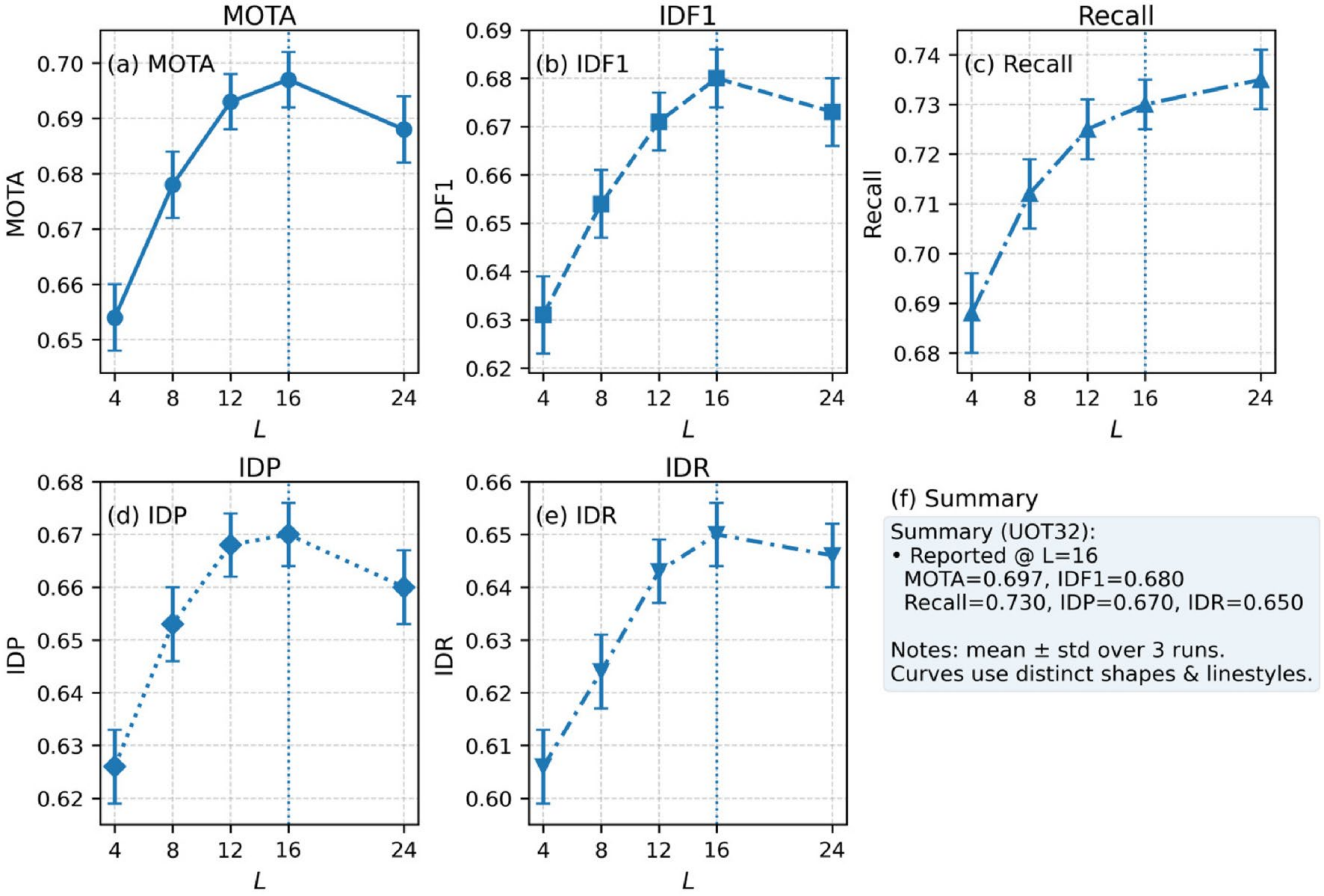
Results of time window hyperparameter sensitivity experiments.

To further investigate the robustness of the proposed spatiotemporal modeling scheme, we conducted a series of hyperparameter sensitivity experiments focusing on temporal modeling configurations. In particular, we varied key parameters such as temporal window length, and attention head configuration to examine their influence on tracking accuracy and association stability. These experiments provide insights into the trade-off between long-term motion awareness and computational efficiency, guiding the selection of optimal settings for practical deployment. First, the experimental results of the time window length are given on the UOT32 dataset, and the experimental results are shown in Fig. 11.

From the figure, it can be observed that different time window lengths have a significant impact on the performance of multi-object fish tracking. As the time window increases, the model is generally able to capture long-term motion patterns of targets more effectively, resulting in more stable performance in terms of association accuracy and trajectory continuity. However, when the time window becomes excessively long, the accumulation of redundant information and feature noise can interfere with detection and association, leading to a slight decline in certain metrics. This observation aligns with the principle emphasized in this work of balancing long-term information and immediate responsiveness in spatiotemporal feature modeling.

**Fig. 12:**
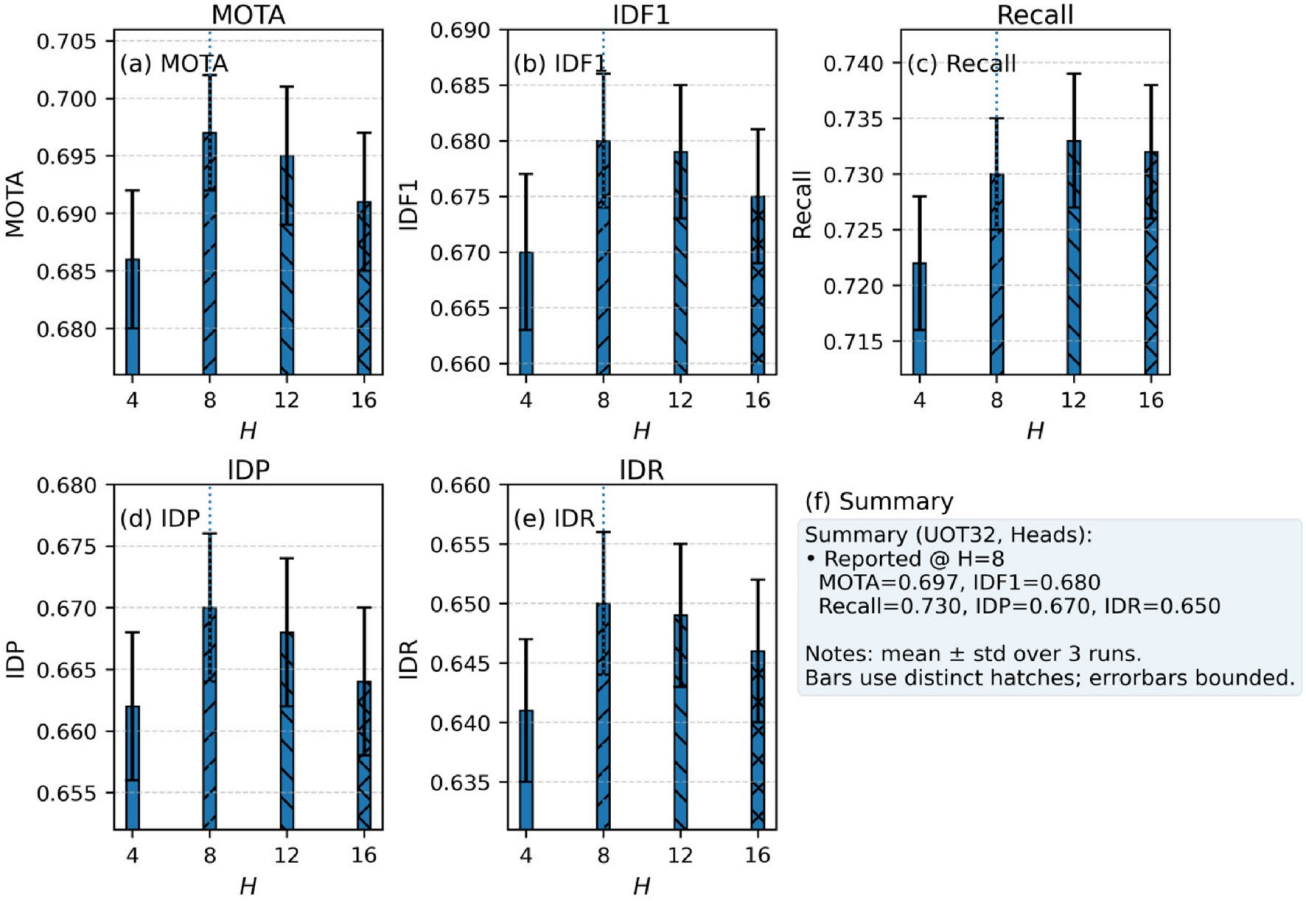
Experimental results on sensitivity of attention head number hyperparameter.

Specifically, a medium-length time window offers advantages in enhancing cross-frame feature consistency and reducing identity switches caused by occlusion, enabling the model to maintain high robustness even in complex scenes. In contrast, shorter time windows, while achieving lower latency, fail to fully utilize historical trajectory information and are more prone to tracking loss in situations with occlusion or dense target distributions. These experimental results validate the effectiveness of the proposed cross-frame encoding and trajectory-aware decoding strategy in temporal modeling, and provide guidance for selecting an appropriate time window during the deployment phase. Furthermore, the experimental results of the attention head are given, as shown in Fig. 12.

From the figure, it can be seen that variations in the number of attention heads exert a relatively moderate influence on the overall performance of multi-object fish tracking. Increasing the number of heads within a certain range can enhance the model’s representational capacity during cross-frame feature alignment. However, it also introduces additional computational overhead and accumulates noise, leading to a slight decline in some metrics once the optimal configuration is exceeded. These results indicate that an appropriate head configuration can achieve a balance between capturing global spatiotemporal dependencies and maintaining operational efficiency, which is consistent with the design objectives of the proposed cross-frame encoding and trajectory-aware decoding strategy.

Specifically, when the number of attention heads is set to a moderate scale, the model exhibits strong performance in both detection accuracy and association stability, suggesting that the multi-head mechanism at this configuration can effectively allocate attention to capture target information across different scales and motion patterns. Conversely, too few heads limit the diversity of feature modeling, while too many may dilute the effective information utilization of each head. This finding provides practical guidance for selecting an appropriate attention head configuration in real-world deployment and further validates the robustness of the model with respect to structural parameter adjustments.

## Conclusion

This paper addresses the challenges of accuracy and stability in multi-object fish tracking under complex underwater scenarios by proposing a unified Transformer framework that integrates cross-frame spatiotemporal encoding with trajectory-aware decoding. In the encoding stage, temporal difference and frame position embeddings, together with a residual motion enhancement mechanism, are employed to effectively improve cross-frame feature alignment and long-term motion pattern modeling capabilities. In the decoding stage, trajectory extrapolation priors and temporal association attention are introduced to explicitly constrain the range of cross-frame feature aggregation, thereby achieving unified optimization of detection and identity association. Evaluations on both our self-constructed dataset and the UOT32 benchmark demonstrate that the proposed method significantly outperforms current state-of-the-art tracking algorithms in key metrics such as MOTA, IDF1, and Recall, while exhibiting superior robustness in occlusion recovery and trajectory continuity. Ablation studies and visualization analyses further validate the effectiveness and complementarity of each module, confirming the overall advantage of our approach for underwater multi-object tracking tasks.

Future work will focus on further enhancing the model’s real-time performance and cross-domain generalization capabilities. Specifically, lightweight optimization strategies such as Transformer structure simplification, model pruning, and knowledge distillation will be explored to reduce inference latency and improve deployment efficiency on edge devices. In addition, adaptive time-window mechanisms and dynamic attention allocation strategies will be introduced, enabling the tracker to automatically adjust association policies according to scene complexity and target motion dynamics. Furthermore, cross-modal fusion with multimodal data–such as sonar imaging and underwater environmental parameters–will be considered to enhance perception under extreme illumination and high turbidity. By combining these improvements with real-time experimental validation, the future work will also conduct comprehensive assessments of the model’s deployment value in practical aquaculture monitoring systems, aiming to achieve efficient, stable, and scalable underwater tracking for intelligent fishery management.

## Data Availability

All data in this study can be obtained by sending an email to the corresponding author
